# Editorial: Biomarkers and mechanisms of gastrointestinal diseases

**DOI:** 10.3389/fgene.2024.1516063

**Published:** 2024-11-06

**Authors:** Zerrin Isik, Gizem Calibasi-Kocal, Omer Yilmaz

**Affiliations:** ^1^ Department of Computer Engineering, Dokuz Eylul University Izmir, Izmir, Türkiye; ^2^ Department of Translational Oncology, Institute of Oncology, Dokuz Eylul University, Izmir, Türkiye; ^3^ Broad Institute of MIT and Harvard, Cambridge, MA, United States; ^4^ Department of Biology, Massachusetts Institute of Technology, Cambridge, MA, United States; ^5^ The David H. Koch Institute for Integrative Cancer Research, Massachusetts Institute of Technology, Cambridge, MA, United States

**Keywords:** gastrointestinal diseases, transcriptome analyses, risk factor analysis, nutrigenomics, biomarker discovery

Investigating and resolving the complicated biology of gastrointestinal diseases has never been more necessary, as early diagnosis and tailored treatments have become the keystone of precision medicine. With gastrointestinal disorders ranging from inflammatory-related conditions to life-threatening situations such as cancers, identifying key biomarkers and understanding the molecular drivers behind these diseases is essential. These contributions improve our awareness about disease initiation, progression, and prognosis and offer translational insights that can configure forthcoming diagnostic and therapeutic strategies. From innovative computational approaches to the discovery of novel biomarkers, this Research Topic provides an opportunity to facilitate more personalized, effective treatments for patients experiencing complex conditions (Marafini and Monteleone, 2021; Ullah et al., 2022; de Back et al., 2024).

As we consider the molecular mechanisms underlying gastrointestinal diseases, the following studies present powerful improvements in biomarker discovery and predictive modeling. Each contribution within this Research Topic highlights exclusive approaches that explore the genetic, epigenetic, and multi-omics views of gastrointestinal-related diseases, shedding light on the complex biological processes that underlie gastrointestinal disease initiation, progression, and treatment. Below, we summarize the key findings of these innovating studies, which collectively indicate the influence of precision in diagnosing and managing gastrointestinal-related conditions.

The study by Sun et al. examined the expression of DDX59-AS1 and its prognostic value in oral squamous cell carcinoma (OSCC). They gathered OSCC samples from The Cancer Genome Atlas (TCGA) database. The differential expression of DDX59-AS1 was calculated between OSCC and healthy samples using the Wilcoxon rank sum test. Logistic regression was applied to show the association between DDX59-AS1 and clinicopathological characteristics. Consequently, T stage, clinical stage, age, and race were all substantially connected with the high expression of DDX59-AS1. Multivariate survival analysis demonstrated that lower overall survival rates were linked to higher DDX59-AS1 expression. In conclusion, this study identified DDX59-AS1 as a significant predictor and treatment target of OSCC.

Sun et al. designed a prognostic model based on cell adhesion genes, that might forecast liver hepatocellular carcinoma (LIHC) metastasis and help with prognosis. The TCGA and GEO databases provided LIHC-related expression data. The Kyoto Encyclopedia of Genes and Genomes (KEGG) database was used to gather genes linked to cell adhesion. The 371 LIHC samples were divided into four subgroups using nonnegative matrix factorization clustering. The PPAR signaling pathway and amino acid metabolism were linked to the 58 differentially expressed genes between four subtypes. The genes IGSF11, CD8A, ALCAM, CLDN6, JAM2, ITGB7, SDC3, CNTNAP1, and MPZ were included in the prognostic signature. The qRT-PCR showed the expressions of nine genes between normal and LIHC tissue. In conclusion, a useful tool for the molecular diagnosis of patient prognosis was presented by the nine genes as a prognostic model.

Dietary factors associated with colorectal cancer (CRC) and inflammatory bowel disease (IBD) are reviewed by Ho et al. Intestinal stem cell (ISC) nutrigenomics is the subject of this review, which examines how particular foods change gene expression to affect ISC activity and function. A combination of preclinical and clinical research indicates that some macronutrients, such as sugar and dietary fat, serve important roles in causing intestinal inflammation and carcinogenesis. However, several micronutrients, like vitamin D and fiber, have anti-inflammatory and anti-tumorigenic properties. Dietary fat activates Wnt/β-catenin and NF-kB, which in turn promote inflammation and carcinogenesis. Because dietary fat activates the Notch signaling pathway, which neither fiber nor vitamin D can reverse, dietary sugar can cause inflammation by inhibiting it. Studies on mice CRC have shown that ketogenic diets have anti-cancer effects, despite the lack of complementary human data. Rodent studies highlighted that a low-calorie diet may reduce the symptoms of IBD. Understanding how particular nutrients and metabolites interact with stem cell regulatory networks should be the primary goal of future studies. The goal of future research should be to clarify the mechanisms by which nutrients and metabolites interact with the regulatory networks of stem cells.

The Ren et al. study conducted a gene risk analysis and emphasized the biological mechanisms of 38 loci related to the susceptibility to ulcerative colitis (UC). They evaluated genes on the FinnGen database’s genome-wide association studies (GWAS) summary statistics of UC using the MAGMA software. To generate UC risk genes, the outcome of the four genetic analysis programs (MAGMA, UTMOST, FUSION, and FOCUS) was combined. To determine the link of causality between the risk genes and UC, Bayesian colocalization and Mendelian randomization (MR) analyses were employed. The same investigations were carried out using a different GWAS data set on IEU to verify the results. PIM3 was suggested as a risk gene for UC as a result of this research.

Collectively, these studies emphasize the worth of biomarker discovery and the clarification of molecular mechanisms in improving the diagnosis, prognosis, and treatment of gastrointestinal diseases. As we resolve the genetic and epigenetic landscapes of these conditions, integrating multi-omics data and computational tools will be decisive in translating these findings into clinical applications. We hope that the contributions in this Research Topic affect further exploration into the biomarkers and mechanisms that describe gastrointestinal diseases, aiming to improve patient outcomes through precision medicine.
